# Enhancing mental health awareness in emergency services (the ENHANcE I project): cross-sectional survey on mental health stigma among emergency services staff

**DOI:** 10.1192/bjo.2021.37

**Published:** 2021-04-12

**Authors:** Cassie M. Hazell, Yasin Koc, Sorcha O'Brien, Sarah Fielding-Smith, Mark Hayward

**Affiliations:** School of Social Sciences, University of Westminster, UK; Department of Social Psychology, University of Groningen, The Netherlands; School of Psychology, University of Sussex, UK; Department of Experimental Psychology, University of Oxford, UK; Research & Development Department, Sussex Partnership NHS Foundation Trust, UK; and School of Psychology, University of Sussex, UK

**Keywords:** Stigma, mental health awareness, police, ambulance, accident and emergency

## Abstract

**Background:**

The number of mental health-related 999 calls to emergency services has increased in recent years. However, emergency services staff have an unfavourable reputation when it comes to supporting people experiencing mental health problems.

**Aims:**

To assess the levels of explicit and implicit mental health stigma among accident and emergency, ambulance and police staff, and draw comparisons with the general population. Additional analyses sought to identify which variables predict mental health stigma among emergency services staff.

**Method:**

A cross-sectional survey of 1837 participants, comprising four independent groups (accident and emergency, ambulance and police staff, and the general population).

**Results:**

Levels of mental health stigma across all four groups were lower than those reported in recent surveys of the general population by the ‘Time to Change’ campaign. Within this study, explicit levels of mental health stigma were lower among the general population compared with emergency services staff. There was no difference between emergency service professions, nor were there any between-group differences in terms of implicit mental health stigma. The only consistent predictors of mental health stigma were attitudes and future behavioural intentions, whereby increased stigma was predicted by increased fear, reduced sympathy and greater intended discrimination.

**Conclusions:**

Our findings suggest that levels of mental health stigma have improved over time, but there is room for improvement in emergency services staff. Interventions to improve mental health stigma may be most effective if, in line with the cognitive–behavioural model of stigma, they target attitudes and behavioural intentions.

People who experience mental health problems tend to have greater contact with emergency services than the general public, for reasons that may directly or indirectly relate to their mental health status. For example, those with mental health problems are more likely to be victims of crime,^1,2^ experience physical health conditions^3,4^ and require immediate health interventions.^5^ Multiple emergency services are also frequently called upon to respond to persons experiencing a mental health crisis.^6^ Despite the frequent contact, emergency services staff have an unfavourable reputation with regard to their treatment of persons with mental health problems,^7,8^ which can deter people experiencing mental health problems from attending hospital^9^ or reporting crimes.^7^ The UK Government has made it a priority for emergency services to improve their working relationship with those experiencing mental health problems, with a particular focus on reducing mental health stigma.^6,10,11^ Several descriptive studies have assessed the rates of mental health stigma among emergency services staff,^12–14^ but few have explored factors that predict stigma.^15^ One proposed explanatory model of stigma posited by Thornicroft et al^16^ utilises a cognitive–behavioural framework. This model suggests that stigma is a product of a lack of knowledge (ignorance), negative attitudes (prejudice) and problematic behaviour (discrimination), and has been endorsed by the National Institute for Health and Care Excellence.^17^ This model is yet to be applied to emergency services staff. Moreover, Thornicroft et al^16^ suggest that the current literature does not focus sufficiently on interventions, and assumes that these attitudes are hard to change. Our aim was to investigate the predictors of stigma, and use this information to inform the selection and development of interventions. As a result, in line with Thornicroft's framework suggesting that ignorance leads to prejudice and discrimination, we used prejudice and discrimination as dependent variables capturing the affective and behavioural components of stigma, and used lack of knowledge (e.g. positive or negative false attributions to mental health) as the predictor of these components. Moreover, we included several demographic variables and current contact as the well-known predictors of stigma, and personal experience and exposure (e.g. professional training) variables to identify possible areas for intervention.

## Research questions

The present study aimed to answer two research questions. First, how do levels of mental health stigma in emergency services staff compare with the general population (including a comparison with data from the UK Government's ‘Time to Change’ (TTC) campaign, and a comparison with an independent general population sample)? Second, what cognitive–behavioural and experience variables predict levels of (both explicit and implicit) mental health stigma in emergency services staff?

## Method

### Design

We utilised a between-group, cross-sectional survey, comprising four participant groups: police, ambulance and accident and emergency (A&E) staff, and members of the general population. These groups were compared in terms of their explicit (i.e. self-reported) and implicit levels of stigma toward people with mental health problems.

### Participants

Participants were either members of the general population (i.e. not working in the emergency services) or emergency services employees (police, ambulance or A&E staff) working in a role that involved the potential to come into direct contact, either in person or via the telephone, with the public. We aimed to recruit 300 participants per group (police, ambulance and A&E staff, and members of the general population), for a total of 1200 participants. The recruitment target is based on the results of an *a priori* power calculation that revealed 268 participants are needed to detect a medium between-group effect (*d* = 0.30) with a power of 0.8 and an *α* of 0.05.

### Procedure

The online survey was promoted via emergency services in South-East England and via social media and survey sharing sites. The promotional materials contained a link to the online survey; upon clicking on the link, participants were presented with information about the study and asked to complete an eligibility assessment. Eligible persons were invited to complete an informed consent statement, followed by a series of self-report questionnaires and a brief computer task. Finally, participants were presented with a debrief statement.

### Measures

#### Sample characteristics

In addition to asking basic demographic questions, we assessed the level of contact participants had with mental health in terms of personal experience (the extent to which participants had their own experience of mental health problems), personal exposure (how much contact participants had with persons experiencing mental health problems in their personal lives), professional contact (the extent to which participants had come into contact with people experiencing mental health problems within their job), professional training (the extent to which participants had received training in supporting people with mental health problems) and confidence (participants’ confidence in their ability to support people with mental health problems). All of the above variables were assessed with a single-item, seven-point Likert scale, with one indicating never and seven indicating very much. We used personal experience, professional training and confidence in our analysis. Profession and personal exposure variables are already captured by the Reported and Intended Behaviour Scale (RIBS) current contact scale.

In the original project protocol, we planned to report the results of the Mental Health Knowledge Schedule (MAKS).^18^ However, we were unable to validate the pre-established factor structure, nor were we able to determine a revised one. We therefore have not included the MAKS in our present analysis. With respect to the other measures used, we have used the factor structures derived from our own analysis.

#### The UK Department of Health Community Attitudes to Mental Illness questionnaire

The UK Department of Health Community Attitudes to Mental Illness questionnaire (CAMI) is the measure used by the TTC campaign to assess mental health stigma. There are multiple versions of the CAMI, and we used the original 27-item version reported by the TTC campaign.^19^ Higher scores indicate greater acceptance of persons with mental health problems (i.e. less stigma). We used the CAMI as the prejudice component of stigma. Within the current study, the scale had high internal consistency (Cronbach's *α* = 0.87).

#### Social Distance Scale

The version of the Social Distance Scale (SDS) used in the present study assessed the extent to which a person wishes to distance themselves from someone with mental health problems.^20^ The SDS has seven items that form a single scale, with higher scores indicating less need for social distance (i.e. less stigma). We used the SDS as the discrimination component of stigma. Psychometric evaluation of data from the current study suggests that the measure has strong internal consistency (Cronbach's *α* = 0.86).

#### Implicit attitudes

In addition to the questionnaires above, we utilised the Brief Implicit Association Test (BIAT) to measure implicit negative perceptions of those with mental health problems.^21^ The BIAT was modified to detect danger-related mental health attitudes. The target categories used were ‘mental illness’ versus ‘physical disability’, with the attribute categories of ‘dangerous’ versus ‘safe’. The time taken for participants to pair a target with an attribute is taken to represent the participant's underlying attitudes. That is, pairing ‘mental illness’ with ‘dangerous’ faster than pairing ‘mental illness’ and ‘safe’ suggests increased levels of stigma. Scores were computed with the algorithm developed by Rüsch et al.^21^ We used BIAT as an implicit prejudice component of stigma.

Participating in the BIAT required participants to press a right-hand response key if the word matched either of the two categories, and a left-hand response key if it did not match either category. There were two blocks of 20 trials each, one with the category pairing mental illness/dangerous and the other one with the pairing mental illness/safe. The blocks were counterbalanced across participants. In each block, the four practice trials were excluded from analyses, as were any participants who incurred >30% errors.

The completion of the BIAT was an optional part of the survey because this required the downloading of the appropriate software. Consequently, the sample size for this part of the analysis is much smaller than for other parts of the survey.

#### Attribution Questionnaire

The Attribution Questionnaire^22^ originally had 20 items assessing beliefs about people with mental health problems. However, our psychometric evaluation suggested the removal of items 7, 13, 15 and 20, and these items were excluded. The Attribution Questionnaire has two subscales: fear toward people with mental health problems and sympathy for people with mental health problems. We used the Attribution Questionnaire as the lack of knowledge component of stigma. Higher scores indicate greater fear or sympathy toward those with mental health problems. The measure has strong internal consistency (fear scale, Cronbach's *α* = 0.92; sympathy scale, Cronbach's *α* = 0.73).

#### RIBS

The RIBS^23^ has eight items that measure either current contact or future behavioural intentions toward those with mental health problems, across four contexts: living with, working with, friends with or neighbours with. We only used current contact as a predictor of attitudes by getting a sum score of four items. Higher scores indicate greater current contact with someone experiencing mental health problems.

### Ethics

The authors assert that all procedures contributing to this work comply with the ethical standards of the relevant national and institutional committees on human experimentation, and with the Helsinki Declaration of 1975, as revised in 2008. All procedures involving human participants were approved by the Health Research Authority (Integrated Research Application Service identifier 224998; Research Ethics Committee reference 17/LO/1536). Written informed consent was obtained from all participants via an online tick-box statement.

### Analysis plan

First, to determine whether levels of mental health stigma reported in our sample differed from those reported previously by the TTC campaign,^19^ we conducted four separate, one-sample *t*-tests comparing our participants’ scores on the CAMI to the mean general population score reported by the TTC campaign (mean 108.9). Second, to assess differences in levels of mental health stigma between our three emergency services groups and our general population sample, we ran three separate, one-way (participant group: police versus ambulance versus A&E versus general population) independent analyses of variance, one for each of three dependent variables: the SDS, CAMI and BIAT. Where any significant main effects were identified, these were further explored with Games-Howell *post hoc* tests. Finally, to identify predictors of mental health stigma in each of the three emergency services staff groups, we ran three separate multiple regression models (one per staff group), with scores on the CAMI as the dependent variable, and the demographic variables (i.e. age, gender, educational level), experience and exposure variables (i.e. personal experience, professional training, confidence and current contact) and the knowledge component of the cognitive–behavioural model of stigma (i.e. fear and sympathy) as the predictors.

## Results

### Sample characteristics

A total of 2579 participants began the survey; however, after excluding those participants who did not provide consent, our final sample size was 1837. The sample characteristics are outlined in Table 1. Across all participant groups, the sample was largely White British and middle-aged, and had some level of formal education. The gender split of our participants varied by group, with a greater number of women for ambulance and A&E staff and the general population, and a greater number of men for police staff.

**Table 1:** Sample characteristics and descriptive statistics for the survey questionnaires

	A&E		Ambulance		Police		General population	
	*n*	*n* (%) or mean (s.d.)	*n*	*n* (%) or mean (s.d.)	*n*	*n* (%) or mean (s.d.)	*n*	*n* (%) or mean (s.d.)
Demographics								
Age	361	40.96 (11.35)	378	35.54 (11.51)	633	39.88 (9.85)	465	35.92 (12.66)
Gender	361		378		633		465	
Male		73 (20.20)		179 (47.40)		332 (52.40)		81 (17.40)
Female		284 (79.70)		195 (52.60)		294 (46.40)		376 (80.90)
Other		2 (0.60)		1 (0.30)		2 (0.30)		6 (1.30)
Prefer not to say		2 (0.60)		3 (0.80)		5 (0.80)		2 (0.40)
Ethnicity	361		378		633		465	
White British		275 (76.20)		338 (89.40)		587 (92.70)		305 (65.60)
White other		35 (9.70)		32 (8.50)		26 (4.10)		82 (17.60)
Asian/Asian British		32 (8.90)		0 (0)		2 (0.30)		32 (6.90)
Black/African/Caribbean/Black British		10 (2.80)		0 (0)		1 (0.20)		12 (2.60)
Mixed ethnicity		1 (0.30)		4 (1.10)		7 (1.10)		18 (3.90)
Other		5 (1.40)		1 (0.30)		3 (0.50)		12 (2.60)
Prefer not to say		2 (0.80)		3 (0.80)		7 (1.10)		4 (0.90)
Education	361		378		633		465	
No formal education		2 (0.60)		3 (0.80)		8 (1.30)		9 (1.90)
Secondary school		33 (9.10)		36 (9.50)		132 (20.90)		48 (10.30)
College		44 (12.20)		103 (27.20)		257 (40.60)		134 (28.80)
Undergraduate		182 (50.40)		190 (50.30)		191 (30.20)		153 (32.90)
Postgraduate		97 (26.90)		42 (11.10)		38 (6.00)		120 (25.80)
Prefer not to say		3 (0.80)		4 (1.10)		7 (1.10)		1 (0.20)
Stigma								
SDS	361	2.92 (0.55)	378	3.01 (0.54)	633	3.02 (0.54)	465	3.19 (0.58)
CAMI	361	115.17 (14.95)	378	114.51 (13.77)	633	114.34 (13.21)	465	118.69 (14.01)
Implicit attitudes	40	0.42 (0.45)	65	0.32 (0.43)	68	0.30 (0.46)	75	0.25 (044)
Predictors								
Attribution Questionnaire								
Sympathy	361	6.51 (1.58)	378	5.98 (1.32)	633	6.25 (1.42)	465	6.61 (1.46)
Fear	361	2.43 (1.24)	378	2.35 (0.95)	633	2.20 (0.99)	465	2.04 (1.03)
RIBS								
Current behaviour	356	2.38 (1.16)	374	2.43 (1.23)	632	2.66 (1.03)	462	2.53 (1.13)
Intended behaviour	360	4.17 (0.78)	374	4.20 (0.66)	632	4.33 (0.67)	465	4.45 (0.69)
Experience and exposure								
Personal experience	361	2.89 (2.12)	378	3.75 (2.01)	633	4.35 (2.25)	465	4.56 (2.31)
Personal exposure	361	4.32 (2.12)	378	4.53 (1.89)	633	5.06 (1.89)	465	5.34 (1.85)
Professional contact	361	5.60 (1.71)	378	6.30 (1.18)	633	6.14 (1.43)	465	4.35 (2.25)
Professional training	361	3.53 (1.88)	378	3.99 (1.57)	633	3.74 (1.79)	465	2.86 (2.22)
Confidence	361	4.27 (1.63)	378	4.33 (1.31)	633	4.88 (1.43)	465	4.40 (1.84)

A&E, accident and emergency; SDS, Social Distance Scale; CAMI, The UK Department of Health Community Attitudes to Mental Illness questionnaire; RIBS, Reported and Intended Behaviour Scale.

### How do levels of mental health stigma compare with TTC data?

All of our participants reported significantly less stigma than the 2014 TTC sample (A&E staff: *t*(360) = 7.96, *P* < 0.001; ambulance staff: *t*(377) = 7.92, *P* < 0.001; police staff: *t*(632) = 10.37, *P* < 0.001; general population: *t*(464) = 15.07, *P* < 0.001).

### How do levels of mental health stigma in emergency services staff compare with those observed in the current general population sample?

There was also a significant main effect of participant group on attitudes toward people with mental health problems, as measured by the CAMI (*F*(3, 1833) = 10.31, *P* < 0.001). *Post hoc* tests revealed that the levels of mental health stigma were significantly greater in emergency services staff (A&E staff: *P* = 0.003; ambulance staff: *P* < 0.001; police staff: *P* < 0.001) compared with the general population. All other *post hoc* comparisons were non-significant (*P*s ≥ 0.82).

There was a significant main effect of participant group on the extent to which participants wished to distance themselves from people with mental health problems, as measured by the SDS (*F*(3, 1833) = 18.48, *P* < 0.001). Similar to the results found with CAMI, the general population were significantly lower in mental health stigma than A&E staff (*P* < 0.001), ambulance staff (*P* < 0.001) and police staff (*P* < 0.001), meaning that mental health stigma was greater in emergency services staff compared with the general population. All other *post hoc* comparisons were non-significant (*P*s > 0.05).

There was no significant effect of participant group on implicit stigma attitudes (*F*(3, 244) = 1.23, *P* = 0.30).

### What factors predict levels of explicit mental health stigma in emergency services staff?

#### A&E staff

For A&E staff, the overall regression model was significant (*F*(9, 339) = 31.46, *P* < 0.001), with cognitive–behavioural and experience factors explaining a substantial amount of variance in mental health stigma (*R^2^* = 46%). Reduced mental health stigma was significantly predicted by being female, having higher education, personal experience of mental health issues, current contact, increased sympathy-related beliefs and reduced fear-related beliefs (Table 2). Age, training and confidence did not significantly predict mental health stigma.

**Table 2:** Cognitive–behavioural predictors and their association with positive mental health attitudes, according to participant group

	Accident and emergency		Ambulance		Police	
	*b* [s.e.]	*β*	*b* [s.e.]	*Β*	*b* [s.e.]	*β*
(Constant)	3.74 [0.15]		4.25 [0.16]		4.10 [0.12]	
Demographics						
Age	0.00 [0.00]	0.04	−0.01 [0.00]	−0.10*	0.00 [0.00]	0.03
Gender (female, 0; male, 1)	−0.16 [0.05]	−0.13**	−0.09 [0.04]	−0.09*	−0.01 [0.03]	−0.11***
Educational level	0.06 [0.02]	0.12**	0.04 [0.02]	0.07	0.03 [0.02]	0.06*
Experience and exposure						
Personal experience	0.03 [0.01]	0.14**	0.01 [0.01]	0.03	0.03 [0.00]	0.15***
Professional training	0.01 [0.01]	0.04	−0.01 [0.01]	−0.03	0.00 [0.00]	0.01
Confidence	−0.01 [0.02]	−0.04	−0.01 [0.02	−0.02	−0.00 [0.01]	−0.01
Current contact	0.05 [0.02]	0.11**	0.10 [0.02]	0.27***	0.03 [0.01]	0.06^†^
Attribution Questionnaire						
Fear	−0.19 [0.02]	−0.50***	−0.25 [0.02]	−0.52***	−0.24 [0.01	−0.54***
Sympathy	0.08 [0.01]	0.27***	0.08 [0.01]	0.22***	0.06 [0.01]	0.19***

* *P* < 0.05, **P < 0.01, ***P < 0.001, ^†^*P* = 0.058.

#### Ambulance staff

The regression model was significant for ambulance staff (*F*(9, 356) = 37.32, *P* < 0.001), explaining a similar proportion of the variance in mental health stigma to the preceding regression analysis (*R^2^* = 49%). Reduced mental health stigma was associated with being younger, being female, current contact, increased sympathy-related beliefs and reduced fear-related attitudes. Education level, personal experience, training and confidence did not significantly predict mental health stigma.

#### Police staff

Finally, for police staff, the overall regression model was also found to be significant (*F*(9, 611) = 53.03, *P* < 0.001), and explained a similar amount of variance to the previous models (*R^2^* = 44%). Reduced levels of mental health stigma were significantly predicted by being female, having higher education, personal experience of mental health issues, increased sympathy-related beliefs and reduced fear-related attitudes. Age, current contact, training and confidence did not significantly predict levels of mental health stigma.

### What factors predict levels of implicit mental health stigma in emergency services staff?

Since there were no group differences in implicit stigma (and we only had 172 participants in total who completed the implicit measure), we examined the predictors of implicit stigma for all emergency services staff together. The overall model was significant (*F*(9, 162) = 2.04, *P* = 0.038), and explained a small amount of variance (*R^2^* = 10%). The only significant predictor was age (*b* = −0.01, s.e. = 0.003, *β* = 0.216); older people had more negative implicit stigma. Gender was also marginally significant (*b* = −0.14, s.e. = 0.071, *β* = −0.15, *P* = 0.58); men had more negative implicit stigma.

## Discussion

The aim of this study was to assess the prevalence and predictors of mental health stigma among the general public and emergency services staff. Utilising explicit, self-report measures, we found that levels of mental health stigma across all participant groups were improved relative to those reported by the TTC campaign in 2014,^19^ but that stigma levels among emergency services professionals were significantly higher compared with the general public, with no significant differences apparent between professions. In contrast, our implicit assessment of mental health stigma indicated no significant differences between the four participant groups. The consistent predictors of mental health stigma across emergency services professions were gender and knowledge, whereby reduced stigma levels were predicted by being female, having increased sympathy-related beliefs and having reduced fear-related attitudes. Additionally, reduced stigma was predicted by educational level and personal experience for A&E and police staff, and by current contact for A&E and ambulance staff.

All of our participant groups reported significantly less mental health stigma than the TTC campaign^19^ sample, including the data from our general population sample. This finding likely represents an improvement in mental health stigma over the 5-year period since the reporting of TTC findings. The longitudinal TTC campaign data supports this explanation, as stigma scores improved almost every year from 2008 to 2014.^19^ The longitudinal improvements in mental health stigma could be attributed to the increased exposure to anti-stigma social marketing campaigns,^24^ an overall greater awareness of TTC^25–27^ or more positive representations of mental health in the media and by celebrities.^28^ Further investigation is needed to identify which of these factors may explain our findings.

Within our current sample, we found a significant difference between the stigma levels of emergency services staff and the general population, with stigma levels being higher among A&E, ambulance and police staff. This finding is somewhat surprising, considering the high degree of contact emergency services staff have with persons experiencing poor mental health,^6^ and that increased contact is associated with reduced stigma.^29^ Although we found that current contact predicted lower stigma levels for A&E and ambulance staff, we propose that a more nuanced exploration of contact may be needed in further research, taking into consideration the nature of the contact that emergency service staff may have with those experiencing mental health problems. That is, emergency services staff are having contact with mental health patients largely when their symptoms are at their most severe, in an environment characterised as frantic, time-pressured and risk-focused.^11^ A working environment that is not conducive to providing mental health support, combined with the lack of opportunity for emergency services to be involved in any kind of resolution for the patients, can create feelings of frustration and hopelessness on the part of emergency services staff toward those with poor mental health.^11^ Further increases in contact *per se* are therefore unlikely to improve stigma among emergency service staff. Instead, contact with people with mental health problems would need to take place in atypical contexts that are facilitative of learning (e.g. with training environments facilitated by people with lived experience of mental health problems^30^).

Across the emergency services groups, stigma was consistently predicted by gender, as women reported less stigma within explicit and implicit measures. This finding is consistent with the TTC campaign data,^19^ and may have implications for the targeting of any training that is aiming to reduce stigma. Other findings from our regression analysis provide support for the knowledge component of the cognitive–behavioural model of stigma being important for reduced prejudice and discrimination,^16^ as increased sympathy-related beliefs and reduced fear-related attitudes were consistent predictors of less stigma across the groups of emergency services staff.

### Limitations

Our findings suggest that levels of mental health stigma were generally low among the general public and emergency services participants. This result may be genuine or may reflect a sampling bias. That is, because we did not survey the whole population, it is possible our self-selected sample largely represents only those that are low in mental health stigma. This limitation could be addressed by using audit methods. Also, our questionnaire results could reflect a social desirability bias.^31^ There is some suggestion that this might be the case, as we did not find any significant between-group effects when using implicit measures of mental health stigma. However, the implicit analysis only included a subset of our participants, and this could explain the different results found. Finally, our emergency services participants were all based in South-East England. Mental health stigma levels vary according to region in the UK.^26^ It is therefore reasonable to assume that our results may differ if the study was replicated in a different geographical location.

### Implications

Our findings require replication in other localities, preferably using audit methods. Among our emergency services participants, although levels of mental health stigma were significantly higher than the general public, they were lower than those found by the TTC campagin.^19^ This finding indicates that stigma levels among emergency services staff are improving, but there is room for further improvement. The regression analysis reported here provides a number of targets by which mental health stigma could be improved; for example, enhancing sympathy-related beliefs and reducing fear-related attitudes could improve overall stigma levels. Any interventions or training initiatives aimed at reducing mental health stigma among emergency services staff may be most effective if they are based on the cognitive–behavioural model of stigma.^16^

## Data Availability

The data that support the findings of this study are available from the corresponding author, M.H., upon reasonable request.
